# Balloon Dilatation of a Case of Tuberculous Tracheobronchial Stenoses during the Course of Antituberculous Treatment

**DOI:** 10.1155/2015/618394

**Published:** 2015-03-19

**Authors:** Shimaa Nour Moursi Ahmed, Potjanee Korrungruang, Hideo Saka, Gyo Asai, Yuko Ise, Chiyoe Kitagawa, Masahide Oki

**Affiliations:** ^1^Department of Respiratory Medicine, National Hospital Organization Nagoya Medical Center, Nagoya, Aichi 460-0001, Japan; ^2^Pulmonary Unit, Department of Medicine, Rajavithi Hospital, Bangkok 10400, Thailand; ^3^Department of Respiratory Medicine, Aichi Cancer Center Aichi Hospital, Okazaki, Aichi 444-0011, Japan

## Abstract

We report a case of posttuberculosis (TB) tracheobronchial stenoses presented with progressive exertional dyspnea during the course of anti-TB treatment. An 83-year-old Japanese man was admitted for progressive dyspnea; chest X-ray and CT showed stenosis of distal trachea and left main bronchus. Pulmonary function test revealed reduction of FEV1. Balloon dilatation without stent insertion was the choice for this patient for multiple reasons with marked improvement of symptoms.

## 1. Introduction

Endobronchial TB (EBTB) is defined as TB infection of tracheobronchial tree with microbial and histopathological evidence [1]. Incidence of EBTB is 10–40% of pulmonary TB [2, 3], and incidence of TB bronchostenosis is up to 68% and it is considered the most common cause of tracheobronchial stenosis in Asian countries. It is more common in females and commonly affects left main bronchus [1, 4]. We discuss the reason of balloon dilatation choice as the line of treatment in this case of TB tracheobronchial stenoses.

## 2. Case Presentation

**Figure 1:**
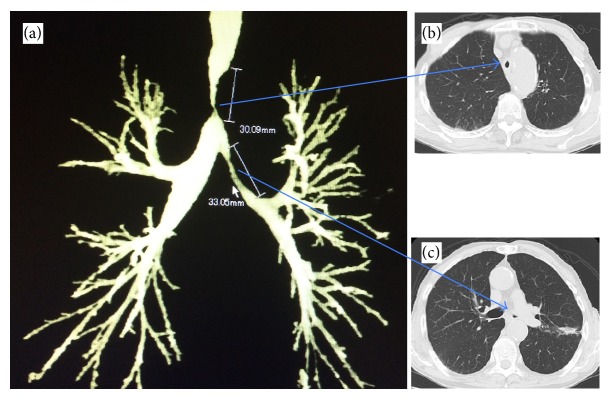
(a) Three-dimensional chest computed tomography (CT) showing stenoses of distal trachea and left main bronchus. (b) Stenosis of distal trachea. (c) Stenosis of left main bronchus.

**Figure 2:**
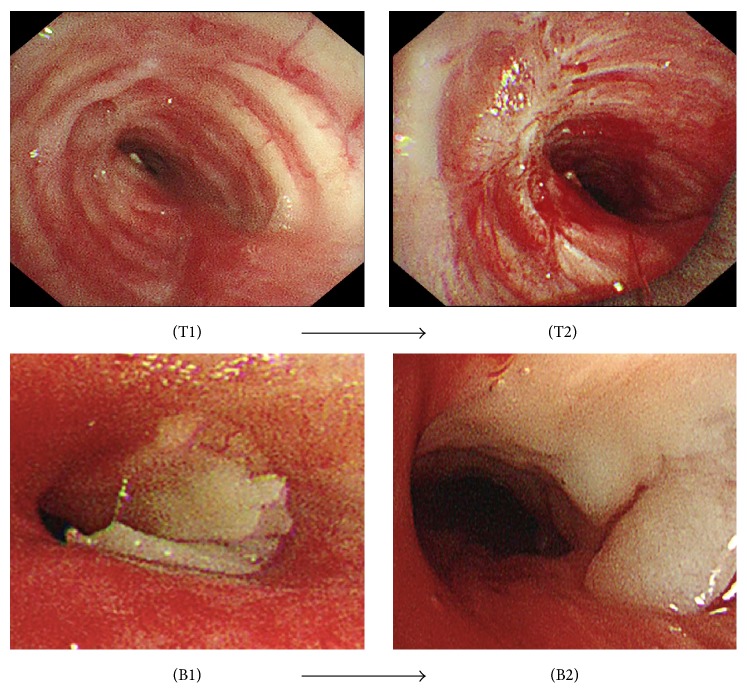
(T1) Distal tracheal stenosis, before ballooning. (T2) After ballooning. (B1) Left main bronchus stenosis, before ballooning. (B2) After ballooning.

We present a case of 83-year-old male with previous cerebrovascular disease, coronary artery bypass surgery, and prostate cancer. He was diagnosed with smear positive pulmonary TB. He received anti-TB 4-drug combination (HREZ). After 2 months of treatment, the patient sputum was converted to negative smear, but he was suffering from progressive dyspnea on exertion with a Medical Research Council (MRC) Dyspnea Scale grade 5 [5]. On the physical examination, wheezing was demonstrated on chest auscultation and O_2_ saturation was 96% in room air. Methylprednisolone sodium succinate 80 mg/day for one week followed by oral prednisolone 40 mg/day was administered for relief of dyspnea, but they were not effective. Chest X-ray demonstrated narrowing of distal trachea and minimal infiltrations in left lower lobe. Chest CT revealed stenosis of distal trachea and left main bronchus; the stenotic part in distal trachea was 5 mm in diameter at the narrowest portion and 30 mm in length. Additionally, the stenosis in left main bronchus was 3 mm in diameter and 33 mm in length (Figure 1). The pulmonary function test indicated a reduction in the percent predicted FEV1 (49.8%). He was referred to Nagoya Medical Center to perform pulmonary intervention. Under general anesthesia, rigid and flexible bronchoscopies were used to perform balloon dilatation under fluoroscopic guidance for both distal trachea and left main bronchus. Tracheobronchial stenosis was of fibrostenotic type. Through the instrument channel of flexible bronchoscope, a controlled radial expansion balloon (Boston Scientific, Boston, MA, USA) was inserted into distal trachea. The diameters used were calculated from the CT taken before the procedure and modified by the real situation at the site. It was inflated to size of 7 mm in diameter for 2 minutes (min) once, 8 mm for 2 min once, 10 mm for 2 min twice, and lastly 12 mm for 4 min twice. Then the balloon was inserted in the left main bronchus and inflated to size of 6 mm for 1 min once, followed by 7 mm for 1 min once (Figure 2). It was sufficient to relieve symptoms with an MRC grade 3. He was discharged two days after the procedure. He resumed his daily activities without oxygen supplementation and there was an improvement of the percent predicted FEV1 (59%). Although the patient did not want to take bronchoscopic follow-up, his condition was reported to be stable in 6 months.

## 3. Discussion

Endobronchial stenosis is one of the main causes of incurable pulmonary TB as it leads to pulmonary closure and resistance to treatment. Severe bronchostenosis often occurs prior to, during, and following treatment of endobronchial TB. It leads to atelectasis of the lung, which limits efficacy of anti-TB treatment. Independent predictors of TB tracheobronchostenosis are as follows: age over 45 years, pure or combined fibrostenotic subtype, and duration from onset of symptoms to initiation of anti-TB drugs more than 90 days [6]. Our patient has 2 of 3 factors, which will make us predict restenosis in the near future.

The mechanism underlying airway stenosis in EBTB was explained in several ways: destruction of bronchial cartilage by caseous necrosis, cicatricial annular stricture due to fibrosis, mural tuberculoma occluding the bronchial lumen, and intramural caseous material [7–10]. The last two lesions are frequently observed in active EBTB [11, 12]. After effective anti-TB chemotherapy, intramural inflammation and caseous material are replaced by fibrosis, which results in stricture of the bronchial lumen. In addition, the presence of peribronchial inflammation, such as with lymphadenitis, would aggravate fibrosis. Ballooning may be a safe and effective approach for dilatation of the narrow bronchus by stretching and expanding the fibrotic tissue within the bronchial wall.

In spite of the conversion of sputum direct smear to negative for acid fast bacilli, the process of EBTB was still going on and stenosis occurred. Authors have explained this low yield because of mucus entrapment by proximal bronchial granulation tissue and further suggested that a negative sputum smear does not preclude the diagnosis of EBTB [13].

Corticosteroids have been used empirically in the treatment of tuberculosis in an attempt to prevent fibrosis. However, the value of using corticosteroids for EBTB is uncertain[14]. Though literature reporting that steroid addition did not help with improvement or clinical healing[4, 15, 16], there is literature arguing that oral or inhaled steroids affect improvement and clinical healing positively in some types of EBTB [17–19]. Corticosteroids are likely to be beneficial in earlier stages when hypersensitivity is the predominant mechanism but are unlikely to be helpful in more advanced cases when extensive fibrosis is present.

Several techniques can be used for treatment of TB tracheobronchostenosis including surgical resection, cryotherapy, argon plasma coagulation (APC), laser photoresection, balloon dilatation, and stenting [20]. Balloon dilatation has several advantages over other methods. It is (1) simple, (2) rapid, (3) well tolerated, (4) minimal invasive and (5) can be done under general or local anesthesia. Also it provides (6) immediate symptomatic relief, and (7) increase of airway dimensions with improvement of lung function [21].

Stent insertion in case of TB tracheobronchostenosis is indicated in cases not suitable for surgery due to patient condition or complexity of stricture, in patient refractory to balloon dilatation, in tracheobronchomalacia, and to seal the fistula [22]. We usually avoid metallic stent especially the uncovered type because of tissue hyperplasia through mesh of the stent and the difficulty to be removed. Silicone stent is more preferred than metallic stent because of easy placement and removal, but it has complications like migration and retained secretions [23].

Ablative techniques are frequently used to reestablish airway patency including heat and cold therapies. Cryotherapy using video-bronchoscopy also exhibits favorable effects but it is a very slow-functioning procedure and needs to be repeated several times per month. It gives better results if combined with balloon dilatation [24]. When APC is used for treatment of TB stenosis, granulation tissue grows repeatedly leading to undesirable effects. It may be treated by electric coagulation, cryotherapy, and balloon dilation [25].

The follow-up study is important to observe the airway patency during and after the treatment of antituberculous drugs. Although this patient did not want to take bronchoscopic follow-up, he has stable clinical condition during 6 months after ballooning without any additional intervention. It was recommended to follow up 3, 6, and 12 months after ballooning, even if the lesion does not recur [26].

In summary ballooning dilatation was the proper choice in this case. At first, the presence of multiple sites of stenosis made the stent insertion difficult. Second, as the patient was still receiving anti-TB treatment, the process of TB stenosis was still on-going. Third, the use of airway stents for benign disease has multiple risks including migration, mucostasis, and formation of granulation tissue. Our aim was to keep the airways patent and relieve symptoms during the course of anti-TB drugs. Even in the case of recurrence of stenoses, we can treat them by ballooning repeatedly. Stent should be taken into consideration if the case is refractory to repeated balloon dilatation.
